# VEGF pathway targeting agents, vessel normalization and tumor drug uptake: from bench to bedside

**DOI:** 10.18632/oncotarget.6918

**Published:** 2016-01-14

**Authors:** Marlous Arjaans, Carolina P. Schröder, Sjoukje F. Oosting, Urania Dafni, Josée E. Kleibeuker, Elisabeth G.E. de Vries

**Affiliations:** ^1^ Department of Medical Oncology, University of Groningen and University Medical Center Groningen, Groningen, The Netherlands; ^2^ Laboratory of Biostatistics, University of Athens, Athens, Greece; ^3^ Department of Materials Science and Metallurgy, University of Cambridge, Cambridge, UK

**Keywords:** antiangiogenic drugs, blood vessel normalization, tumor drug delivery

## Abstract

Vascular endothelial growth factor (VEGF) pathway targeting agents have been combined with other anticancer drugs, leading to improved efficacy in carcinoma of the cervix, stomach, lung, colon and rectum, ovary, and breast. Vessel normalization induced by VEGF pathway targeting agents influences tumor drug uptake. Following bevacizumab treatment, preclinical and clinical studies have shown a decrease in tumor delivery of radiolabeled antibodies and two chemotherapeutic drugs. The decrease in vessel pore size during vessel normalization might explain the decrease in tumor drug uptake. Moreover, the addition of bevacizumab to cetuximab, or panitumumab in colorectal cancer patients or to trastuzumab in breast cancer patients, did not improve efficacy. However, combining bevacizumab with chemotherapy did increase efficacy in some cancer types. Novel biomarkers to select patients who may benefit from combination therapies, such as the effect of an angiogenesis inhibitor on tumor perfusion, requires innovative trial designs and large clinical trials. Small imaging studies with radiolabeled drugs could be used in the interphase to gain further insight into the interplay between VEGF targeted therapy, vessel normalization and tumor drug delivery.

## INTRODUCTION

Angiogenesis is a hallmark of cancer that enables tumor growth [1]. Vascular endothelial growth factor A (VEGF-A) is a key player in the process of tumor angiogenesis, and the VEGF pathway has therefore been an important focus for anti-cancer drug development [2, 3]. Although angiogenesis is recognized to enable tumor growth, some tumors are capable of growing independent of angiogenesis by vessel co-option [4].

Antiangiogenic treatment blocks the formation of new blood vessels. The initially high hopes of VEGF pathway targeting agents as panacea for treatment of solid tumors have been replaced by a more realistic definition of their role. Single agent activity has been shown in renal cell carcinoma, hepatocellular carcinoma, pancreatic neuroendocrine tumors, soft tissue sarcomas and in colorectal cancer. VEGF pathway targeting drugs have been added to other anticancer drugs to improve their efficacy. This approach has only been successful in carcinoma of the cervix, stomach, lung, colon, rectum, ovary, and breast [5-7]. Improved understanding of the underlying mechanisms could support rational drug combinations.

Preclinical and clinical studies have indicated that anti-VEGF therapy induces changes in function and architecture of existing blood vessels, described as vessel normalization [8]. Major characteristics of vessel normalization are reduced number and size of immature vessels, increased vessel pericyte coverage and reduced interstitial fluid pressure (IFP) [8, 9].

Another consideration is that changes in tumor vasculature caused by anti-VEGF therapy could also affect tumor uptake of other drugs. If preclinical data could be used to predict the behavior of combination therapy, this would be of great benefit in the clinic. At present, however, translation of preclinical antiangiogenesis data to the clinic remains challenging. We reviewed the literature on the interplay between VEGF pathway targeting agents, vessel normalization and tumor drug delivery in the preclinical and clinical setting.

### Vessel normalization and VEGF-targeted agents

The vascular organization and structure of tumors differs from normal tissue [10]. Tumor vasculature is more tortuous and chaotic, with inadequate pericyte coverage, increased breaches between endothelial cells and alternating thick and thin basement membranes. This leads to increased vessel permeability and high IFP causing hypoxia [8, 9] Preclinical studies have shown that anti-VEGF therapy can initiate vessel normalization. Vessel normalization is measured in the preclinical and clinical setting by decreased vessel diameter, blood volume, mean vessel density (MVD), macromolecular permeability, IFP and edema. Vessel normalization leads to an increase in partial oxygen pressure and perivascular cell coverage in the tumor [8, 9, 11-15]. In this review, we defined vessel normalization as pruning and remodeling of abnormal tumor vessels, leading to vessels resembling normal tissue vasculature in terms of structure and function [8, 9].

Translating preclinical insights about vessel normalization to the clinic has been challenging. This is due in part to the differences between tumor-bearing mice and human patients. Murine models with subcutaneous, fast-growing human tumors are generally used [16, 17]. In patients, primary tumor lesions can be located anywhere in the body and usually are slow-growing, with doubling times of months to years compared to weeks in murine models. Even with metastatic disease, clinical progression is generally much slower than in murine models. In addition, preclinical models often comprise a single, subcutaneous human tumor with murine vasculature. These tumors are often treated for weeks, at most. In patients, tumor and vasculature are of human origin, and long-term treatment is required for optimal antitumor effect. Furthermore, normal vasculature in patients will be aged, as for most cancers incidence rates increase with age [18]. To improve translation to the clinic, preclinical studies should ideally be representative for the stage of disease treated in the clinic, consist of tumor cells with a compatible immunocompetent microenvironment and examine combination therapies at appropriate dosages analogous to the clinic [19-21].

At the moment several VEGF pathway targeting agents are available. Registered drugs include small molecule tyrosine kinase inhibitors (TKI) targeting the VEGF receptors (VEGFR), and antibodies targeting VEGF and VEGFR2.

### VEGFR TKIs

A recent meta-analysis evaluated the efficacy and safety of combining VEGFR TKIs with chemotherapy in patients with solid tumors [22]. Data from 24 randomized controlled trials with a total of 8,961 patients was analyzed, with 879 patients participating in axitinib trials, 3,761 in sorafenib trials, 1,970 in sunitinib trials and 2,351 in vandetanib trials. The addition of VEGFR TKIs to chemotherapy increased side effects. There was an increase in any adverse events (relative risk 1.34, 95% confidence interval (CI) 1.20 - 1.50, *P* < .001) and fatal adverse events (relative risk 1.49, 95% Cl 1.16 - 1.90, *P* = .002) [22].

Results from numerous phase 3 trials combining VEGFR TKIs with chemotherapy showed only marginal to no increased antitumor efficacy (Table 1). When combined with chemotherapy in metastatic colorectal cancer (mCRC), neither vatalanib (first- and second-line treatment) nor sunitinib (first-line treatment ) increased progression-free survival (PFS) or overall survival (OS) [23-25]. In the randomized phase 3 HORIZON II trial, combination of cediranib with chemotherapy led to a clinically irrelevant increase of 0.3 months in PFS (HR 0.84, *P* = .012), and had no effect on OS as first-line therapy in mCRC patients [26]. In addition, in metastatic breast cancer sunitinib had no effect on PFS or OS when combined with chemotherapy as first- and second-line therapy [27, 28]. In non-small cell lung cancer (NSCLC), addition of sorafenib to chemotherapy in the first-line had no effect on OS [29, 30]. Combining vandetanib with chemotherapy as second line NSCLC therapy in the randomized phase 3 ZODIAC trial led to an increase in PFS of 0.8 months (HR 0.79, *P* < .0001) [31]. The randomized phase 3 ZEAL trial trial showed a positive trend in PFS, but no significant increase, when vandetanib was combined with chemotherapy in second-line treatment [32]. In the LUME-Lung 1 randomized phase 3 trial, the addition of nintedanib to chemotherapy in the second line increased PFS with 0.7 months (HR 0.79, *P* = .002), but did not increase OS. A beneficial effect of 2.3 months (HR 0.83, *P* = .036) on OS was only seen in the subgroup of patients with a histological defined adenocarcinoma [33].

**Table 1:** Results from phase III trials combining antiangiogenic therapy with chemotherapy or monoclonal antibodies

Phase III trials combining a VEGFR TKI with chemotherapy (CH)				
Tumor type	VEGFR TKI	PFS (months)	OS (months)	Ref
		*TKI+CH vs CH+/−placebo*	*TKI+CH vs CH+/−placebo*	
**Metastatic colorectal cancer**	vatalanib	*7.7* vs 7.6 (NS)	21.4 vs 20.5 (NS)	23
		5.6 vs 4.2 *(P=*.013)	13.1 vs 11.9 (NS)	24
	sunitinib	7.8 vs 8.4 (NS)	20.3 vs 19.8(NS)	25
	cediranib	8.6 vs 8.3 (P=.012)	19.7 vs 18.9 (NS)	26
**Metastatic breast cancer**	sunitinib	8.6 vs 8.3 (NS)	24.8 vs 25.5 (NS)	27
		5.5 vs 5.9 (NS)	16.4 vs 16.5 (NS)	28
**NSCLC**	sorafenib	4.6 vs 5.4 (NS)	10.7 vs 10.6 (NS)	29
		6.0 vs 5.5 (*P=*.008)	12.4 vs 12.5 (NS)	30
	vandetanib	4.0 vs 3.2 (*P* <.0001)	10.6 vs 10.0 (NS)	31
		17.6 vs 11.9 (weeks; NS)	10.5 vs 9.2 (NS)	32
	Nintedanib	3.4 vs 2.7 (P=.0019)	10.1 vs 9.1 (NS)	33
**Metastatic/recurrent cervical cancer**	cediranib (phase II)	8.1 vs 6.7 (*P=* .032)	13.6 vs 14.8 (NS)	34
**Glioblastoma multiforme**	Cediranib	125 vs 82 (days; NS)	9.4 vs 9.8 (NS)	35

Phase III trials combining bevacizumab (B) with another monoclonal antibody and chemotherapy (CH)				
Tumor type	Combination therapy	PFS (months)	OS (month)	Ref.
**Metastatic colorectal cancer**	cetuximab + B + CH vs B + CH	9.4 vs 10.4 (*P* .01)	19.4 vs 20.3 (NS)	46
	panitumumab + B + CH vs B + CH	10.4 vs 11.4 (HR1.3) (*P* not sported)	19.4 vs 24.5(HR1.4) (*P* not reported)	47
**HER2 positive breast cancer**	trastuzumab + B + CH vs trastuzumab + B + CH	16.5 vs 13.7(*P* =.07)	Not reported	48
		122 vs 11.I (NS)	Not reported	49
		Not reported	97% vs 96% (NS, 38 mo follow-up)	50

Phase III trials combining ramucirumab (R) with chemotherapy (CH)				
Tumor type	Combination therapy	PFS (months)	OS (months)	Ref.
**Gastric and esophageal cancer**	R vs placebo	2.1 vs 1.3 (*P* <.0001)	52 vs 3.8 (P-:.047)	77
	R + CH vs placebo + CH	4.4 vs 2.9(*P* <0001)	9.6 vs 7.4 (P^−^.017)	78
**NSLC**	R + CH vs placebo + CH	4.5 vs 3.0 (*P* <0001)	10.5 vs 9.1 (*P* .023)	79
**Metastatic colorectal cancer**	R + CH vs placebo + CH	5.7 vs 4.5 (*P=* .0005)	13.3 vs 11.7(P .02)	80
**Metastatic breast cancer**	R + CH vs placebo + CH	9.5 vs 8.2 (NS)	27.3 vs 27.2 (NS)	81

Abbreviations: VEGFR TKI= vascular endothelial growth factor receptor tyrosine kinase inhibitor, B=bevacizumab, CH=chemotherapy, NSLC= non-small cell lung cancer, PFS= progression free survival, OS= overall survival, NS= no significant difference, mo=months, Ref=reference.

Recently, the randomized phase 2 CIRCCa trial in metastatic and recurrent cervical cancer patients showed that the addition of cediranib to chemotherapy improved PFS with 1.4 months (HR 0.58, *P* = .032) compared to placebo. However, the addition of cediranib did not increase OS in these patients [34].

In recurrent glioblastoma multiforme (GBM) patients, combining cediranib with chemotherapy in the randomized phase 3 REGAL trial did not increase PFS and had no effect on OS [35]. A small prospective study measured tumor blood perfusion changes with magnetic resonance imaging (MRI) during cediranib treatment in 30 recurrent GBM patients to evaluate the vascular normalizing effects of cediranib [36]. Tumor perfusion increased in 7, decreased in 11 and remained stable in 12 patients. OS prolonged to 348 days in patients with increased perfusion, compared to 169 or 213 days in patients with respectively stable or decreased perfusion (*P* = .019). In another prospective study, patients with newly diagnosed GBM received 30 mg/day cediranib with chemoradiation (*n* = 40) or chemoradiation alone (*n* = 14) [37]. Cediranib increased perfusion in 20 (50%) patients, decreased perfusion in 10 (25%) patients, and in 10 (25%) patients perfusion remained stable. These changes occurred at day 1 and became stable around day 8. However, in one out of the 14 (7%) control patients, perfusion increased with chemoradiation alone. In the combination group, increased perfusion was associated with a median OS of 26.3 months compared to 17.0 months for patients without increased perfusion (*P* = .028). Based on MRI analyses, these two studies show that cediranib increased perfusion in a subgroup of GBM patients, which correlated with increased survival. These results suggested that increased tumor perfusion by cediranib induced vessel normalization might lead to increased tumor drug uptake and improved outcome in these patients [36, 37]. In addition to these studies, preclinical studies have shown that antiangiogenic therapy could potentiate radiotherapy by improving tumor oxygenation [38]. In randomized studies this did not translate to OS benefit. In newly diagnosed glioblastoma patients 2 randomized phase 3 trials, AVAglio and RTOG0825, studied the effect of the addition of bevacizumab to chemotherapy and radiotherapy. Both trials showed that the addition of bevacizumab did improve PFS (4.2 months in AVAglio (HR 0.64, *P* < .001) and 3.4 months in RTOG0825 (HR 0.79, *P* =.007), but had no effect on OS compared to placebo [39, 40].

Blood flow and perfusion are closely related terms. Strictly speaking blood flow is defined as blood volume per time, whereas perfusion stands for blood volume per time per amount of tissue. This means that blood flow can be high, while perfusion e.g. as expressed per 100 g of tissue is low. Accurate measurement of tumor perfusion is of interest in determining effects of antiangiogenic therapy or evaluating drug delivery. Interesting imaging modalities to measure tumor perfusion in a noninvasive matter are PET and dynamic contrast-enhanced (DCE) CT or MRI. In DCE imaging a contrast agent is given and images made before, during and after injection are necessary to calculate contrast concentrations in the area of interest. In case of PET imaging, the tracer is injected and scans are made to directly measure tracer uptake. During DCE-MRI or CT imaging, contrast agents are used to measure perfusion. These contrast agents are not freely diffusible, thus uptake does not solely represent tumor perfusion. However they also provide insight in vasculature permeability. H_2_^15^O-PET is a freely diffusible tracer, thereby directly illustrating tumor perfusion. Furthermore, there is a linear relation between H_2_^15^O or contrast agent concentration and signal intensity as measured by PET or DCE-CT, which enables direct quantification. There is no linear relationship between contrast agent concentration and signal intensity as measured by DCE-MRI, complicating quantification of tumor perfusion [41 - 43].

**Figure 1:**
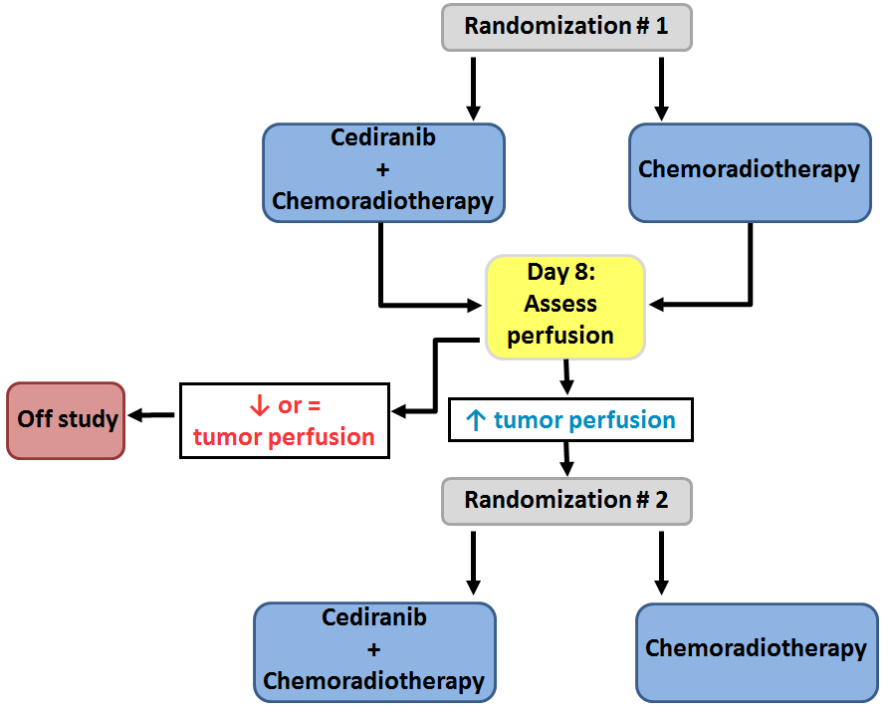
Hypothetical trial to evaluate tumor perfusion as a predictive biomarker for survival in GBM patients To identify the biomarker-positive cohort, initially all patients are randomized to chemoradiotherapy with or without cediranib. In a second phase, only patients with increased perfusion after 8 days on treatment are randomized to continue chemoradiotherapy either with or without cediranib.

To provide insight into the implications of patient selection based on perfusion, we designed a hypothetical trial using an enrichment design for primary GBM patients treated with cediranib and/or standard chemoradiotherapy (Figure 1) [44, 45]. In such a design, the biomarker is evaluated in all randomized patients, but only patients who are defined as biomarker-positive, i.e. patients with increased tumor perfusion after 8 days on initial randomized treatment, are eligible for a second randomization [45]. To identify the biomarker-positive cohort, initially all patients are randomized to chemoradiotherapy with or without cediranib. In a second phase, only patients with increased perfusion after 8 days on treatment are randomized to continue chemoradiotherapy either with or without cediranib. The OS would be compared between randomized arms, to evaluate whether addition of cediranib provides benefit in patients who achieved increased perfusion, irrespective of the initial treatment that led to this increase. Patients with decreased or stable perfusion would be taken off study and complete standard chemoradiotherapy. The second study phase requires 310 patients, to achieve 80% power at a two-sided α of 5%, assuming an OS improvement of 35% (HR = 0.65). This implies that 1,602 patients should have been enrolled upfront in the first randomization, with 460 and 1142 patients initially randomized to treatment with or without cediranib (respectively corresponding to 230 and 80 patients with increased perfusion at 8 days under initial treatment). This is a very large number of GBM patients; the landmark paper demonstrating the additive effect of chemotherapy to first line radiotherapy in GBM required 573 patients, accrued during 20 months in 85 centers in 20 countries [44].

### Bevacizumab

So far, combining another antibody with bevacizumab plus chemotherapy have shown detrimental to modestly beneficial effects (Table 1). For mCRC, combining bevacizumab, the anti-EGFR antibody cetuximab and chemotherapy in the phase 3 CAIRO2 trial did not improve OS compared to bevacizumab with chemotherapy alone. In fact, the addition of cetuximab decreased PFS by 1.2 months (HR 1.22, *P* = .01) [46]. Furthermore, patients receiving cetuximab, bevacizumab and chemotherapy experienced more cetuximab-related side effects. Similar results were obtained in the phase 3 PACCE trial in mCRC patients receiving bevacizumab with chemotherapy or bevacizumab, chemotherapy and the anti-EGFR antibody panitumumab. In the panitumumab group, PFS decreased by 1.4 months (HR 1.27) and no effect was observed on OS [47].

In the phase 3 AVEREL trial in metastatic HER2 positive breast cancer patients, chemotherapy with anti-HER2 antibody trastuzumab or chemotherapy, trastuzumab and bevacizumab was administered [48]. Addition of bevacizumab marginally affected PFS (13.7 months without versus 16.5 months with bevacizumab; HR 0.82, *P* = .07). Another randomized phase 3 trial in metastatic HER2 positive breast cancer patients also showed that addition of bevacizumab to trastuzumab and chemotherapy did not increase PFS (11.1 months without versus 12.2 months with bevacizumab; HR 0.65, *P* = .10) [49]. Furthermore, in the randomized phase 3 BETH trial in early HER2 positive breast cancer patients the addition of bevacizumab to trastuzumab and chemotherapy had no effect on invasive disease free survival and OS at a median follow up of 38 months [50].

It has been suggested that vessel normalization could improve tumor drug uptake. In three small rectal cancer studies, comprising 5, 6 and 32 patients, vessel normalization induced by 5-10 mg/kg bevacizumab was investigated with biopsies, IFP measurements and functional computed tomography (CT) scans. At 12 days after bevacizumab administration, IFP decreased and the fraction of vessels covered with pericytes increased, while the permeability-surface area product remained stable. These findings indicate that bevacizumab induced vessel normalization in these patients [51-53]. However, this does not demonstrate a direct relationship between vessel normalization and improved tumor drug uptake. Imaging with radiolabeled drugs potentially provides a tool to quantify tumor drug uptake. Both preclinical and clinical studies have evaluated the effects of antiangiogenic therapy on tumor drug uptake. At the University Medical Center Groningen, we have developed ^89^Zr-labeled bevacizumab and ^89^Zr-trastuzumab as tracers for positron emission tomographic (PET) scanning to visualize and quantify bevacizumab and trastuzumab biodistribution for preclinical and clinical purposes [54-59]. These tracers can provide insight into how bevacizumab affects uptake of other antibodies. Despite limitations of animal models, these human grade tracers allow translation of preclinical findings to the clinic. In two xenograft models of human HER2-positive ovarian cancer (SKOV-3) and esophageal cancer (OE19) we used PET imaging to evaluate tumor uptake of radiolabeled trastuzumab (SKOV-3 and OE19), IgG (in SKOV-3) and bevacizumab (SKOV-3), before and after bevacizumab treatment [60]. On day 6, after three doses of 5 mg/kg bevacizumab, tumor uptake decreased by 41% and 39% for trastuzumab in SKOV-3 and OE19, respectively. For radiolabeled IgG and bevacizumab, tumor uptake decreased by 28% and 44% respectively after bevacizumab treatment. These results indicate that bevacizumab treatment affected antibody tumor uptake negatively. Bevacizumab therapy reduced microvessel density (MVD) in tumors and increased vessel pericyte coverage, indicating both anti-vascular and vessel normalizing effects of bevacizumab.

Two other preclinical studies reported similar results [61,62]. One 10 mg/kg dose of cross-reactive anti-VEGF antibody B20-4.1 decreased tumor trastuzumab uptake by 50% after 2 days in a xenograft HER2-positive breast cancer model (KPL-4) [61]. Moreover, 10 mg/kg bevacizumab decreased tumor cetuximab uptake by 40% after 4 days in EGFR-positive breast cancer xenograft models (SUM149 and SKBR3) [62].

Importantly, these findings are supported in the clinical setting. A study in patients with renal cell carcinoma (*n* = 11) showed a 47% decrease of ^89^Zr-bevacizumab tumor uptake 2 weeks after one therapeutic infusion of 10 mg/kg bevacizumab [63]. Thus, vessel normalization induced by bevacizumab seems to impair tumor delivery of antibodies.

In addition, two clinical imaging studies suggest bevacizumab also affects tumor drug delivery of chemotherapeutic drugs. In NSCLC patients (*n* = 10), a single dose of 15 mg/kg bevacizumab reduced ^11^C labeled-docetaxel tumor delivery by 22% after 5 hours and by 34% after 4 days [64]. Moreover, tumor drug delivery of ^18^F-5-fluorouracil decreased by 20% at 24 hours after a single administration of 7.5 mg/kg bevacizumab in mCRC patients (*n* = 5) [65]. Phase 3 trials combining bevacizumab with chemotherapy show varying results. Bevacizumab combined with chemotherapy in colorectal, ovarian, cervical and HER2-negative breast cancer has shown an increase in PFS [66 - 70].

A small imaging study evaluated the effect of bevacizumab on tumor perfusion and survival in 36 NSCLC patients [71]. Patients received a dose of 15mg/kg bevacizumab as induction therapy, which after 14 days was followed by the combination of carboplatin, nab-paclitaxel and bevacizumab for a maximum of 6 cycles of 21 days. Blood flow, blood volume and permeability surface, measured by CT, all decreased after induction and during combination therapy. In addition, mean transit time (MTT), a measurement for perfusion, showed a slight increase during combination therapy. This reflects a decrease in perfusion and was suggested to be associated with shorter survival in these patients (*P* = .05). Together with the cediranib imaging studies in GBM patients, these results suggest that perfusion could be a potential read-out for vessel normalization and a possible predictive biomarker for survival [36, 37. 71].

### VEGFR2 antibodies

Intravital imaging showed that DC101, an antibody against mouse VEGFR2, induced vessel normalization in an orthotopic mammary tumor model [72]. This particular study is a key paper as it provided insight into the effect of vessel normalization on pore size of tumor vasculature. The nanoparticles used were quantum dots coated with polymeric imidazole ligand (PIL) (ø = 12 nm) or polyethylene glycol (PEG) (ø = 60 and 120 nm) [73]. Pore size was determined by modeling nanoparticle penetration rate, given as transvascular flux per unit vascular surface area. Vessel normalization by DC101 coincided with a decrease in pore size of tumor vasculature, resulting in an increase of the penetration rate of small nanoparticles (12 nm) but no difference in penetration rate for 60 and 120 nm size nanoparticles. This means that the effect of DC101 on pore size was mainly based on the difference in the transvascular flux of 12 nm particles in tumors with and without DC101 treatment. Moreover, in the E0771 xenograft model treated with DC101, for example, a large spread in transvascular flux of 12 nm nanoparticles was already present in this group (from 0.05 - 0.3 μm s^−1^). Such a substantial variation leads to a large uncertainty in model outcomes on pore size, which was not discussed by the authors. Since antibodies are approximately 12 nm in size, similar to the small nanoparticles, it was suggested that DC101-induced vessel normalization may also improve tumor drug delivery of antibodies [74]. However, although their size is similar, there are substantial chemical differences between nanoparticles used in this study and antibodies, which may affect penetration rate [72, 73]. Firstly, nanoparticles are spherically shaped, whereas antibodies are Y-shaped [75, 76]. Secondly, the mass (density) of the nanoparticles and antibodies may be different. Thirdly, the chemistry of the outer shell of the nanoparticles used in this study is different than the chemistry of antibodies [72]. The PIL-coated 12 nm size nanoparticles have mainly methoxy (R-O-CH_3_) functional end groups in the outer shell, and the PEG-coated 60 and 125 nm particles have hydroxyl (R-OH) functional end groups. On the other hand, antibodies, which can be seen as a combination of four biopolymers, mainly have R-NH_2_ and R-COOH end groups [75, 76]. These differences can make it difficult to translate results from these specific nanoparticles to “nanomedicines” such as antibodies.

This important study also investigated whether DC101-induced vessel normalization could improve efficacy of small chemotherapeutics [72]. Mice were treated with DC101, placebo, DC101 or placebo plus abraxane (albumin-bound paclitaxel, ø = 10 nm) and DC101 or placebo plus doxil (liposome-encapsulated doxorubicin, ø = 100 nm). Tumor doubling times were used as read-out for efficacy. Both DC101 alone and DC101 plus doxil had no effect on tumor doubling times compared to placebo or doxil plus placebo. However, DC101 plus abraxane did increase tumor doubling times compared to placebo plus abraxane. From these data it was concluded that the vessel-normalizing effects of DC101 increased tumor penetration of abraxane. The results of control experiments in this article showed a large range in tumor doubling times in the placebo plus doxil group, which might have influenced the results for this group.

In the clinic, VEGFR2 antibody ramucirumab, which is administered at much lower doses than in the mouse model, has shown very modest or no effect in 5 phase 3 clinical trials (Table 1). Ramucirumab monotherapy (8 mg/kg every 2 weeks) increased PFS by 0.8 months (HR: 0.48, *P* < .0001) and prolonged OS by 1.4 months (HR: 0.77, *P* = .047) in gastric cancer and esophageal junction adenocarcinoma patients in the second line [77]. Adding ramucirumab (8 mg/kg every 2 weeks) to paclitaxel increased PFS from 2.9 months to 4.4 months (HR 0.63, *P* < .0001) and prolonged OS by 2.3 months from 7.4 to 9.6 months (HR 0.80, *P* = .017) in advanced gastric and esophageal junction adenocarcinoma patients [78]. In addition, in NSCLC patients, 10 mg/kg ramucirumab every 3 weeks combined with docetaxel increased PFS by 1.5 months (HR 0.76, *P* < .0001) and prolonged OS by 1.4 months (HR 0.86, *P* = .023) as second-line therapy [79]. In mCRC, FOLFIRI combined with 8 mg/kg ramucirumab prolonged OS compared to FOLFIRI with placebo with 1.6 months from 11.7 to 13.3 months (HR 0.85, *P* = .02) in the second line [80]. However, in breast cancer patients, 10 mg/kg ramucirumab every 3 weeks combined with docetaxel did not affect PFS or OS [81]. The FDA recently approved ramucirumab for advanced gastric and esophageal junction adenocarcinoma, metastatic NSCLC and mCRC patients.

## DISCUSSION

To date, preclinical studies have shown that antiangiogenic therapy can induce vessel normalization. Clinical studies have illustrated that this is not just a preclinical phenomenon; it also occurs in patients. Although some studies have suggested that vessel normalization can improve drug delivery of chemotherapy and enhance efficacy of combination therapies, there is no direct evidence for better tumor drug uptake. On the contrary, both preclinical and clinical studies with radioactive labeled drugs have shown decreased tumor delivery of antibodies as well as chemotherapeutic agents. In the case of chemotherapy, this did not inevitably result in the absence of an additional effect of combination therapy, although it may explain the disappointing results and lack of synergism. In addition to vessel normalization, other mechanisms have been proposed to explain how antiangiogenic agents can improve efficacy of chemotherapy. Certain chemotherapeutic agents can have a local antiangiogenic effect by affecting endothelial cells. This can result in an increased mobilization of circulating endothelial progenitor cells, again promoting tumor angiogenesis. The addition of an antiangiogenic agent can thus counteract this response, thereby improving efficacy of chemotherapy. Furthermore, in response to chemotherapy VEGF and VEGF-receptor expression by tumor cells can increase. The addition of an antiangiogenic agent can subsequently enhance the anti-proliferative action of chemotherapy. Another mechanism proposed is that antiangiogenic agents can inhibit tumor cell repopulation in between chemotherapy cycles, thereby increasing efficacy [82-86].

In the case of antibodies, results from clinical trials in colorectal and breast cancer patients are in line with reduced antibody uptake after antiangiogenic therapy. A possible explanation for decreased uptake could be the change in vessel pore size during vessel normalization. This might influence tumor drug uptake, depending on the size, shape and chemical structure of the drug. New data from a different angle are in line with this interpretation. Wong *et al.* performed a study in NSCLC and pancreatic ductal adenocarcinoma tumor models with low-dose cilengitide, an antiangiogenic agent selectively inhibiting α_ν_ integrins, and verapamil [87]. The combination therapy led to an increase in blood flow and perfusion, MVD and vascular permeability in these tumors.

Moreover, addition of gemcitabine (75 mg/kg) or cisplatin (6 mg/kg) to the combination therapy reduced tumor growth and progression compared to placebo, gemcitabine or cisplatin treatment alone. High-performance liquid chromatography-mass spectrometry showed that cilengitide combined with verapamil increased intratumoral drug delivery of gemcitabine. Overall these findings indicate that tumor angiogenesis or even vascular promotion therapy could improve drug delivery.

Most research concerning vessel normalization has been performed on primary tumors. Preclinical and clinical studies have shown that vessel normalization is a delicate process, occurring during a certain timeframe and dependent on the dose of the antiangiogenic drug [9]. However, it remains unclear how vessel normalization will occur in the metastasized setting. In normal healthy tissue, tissue-specific vessel functions illustrate vessel heterogeneity for different organs [88]. This also applies to the tumor vasculature, and indeed, different types of tumor blood vessels have been identified [89, 90]. In NSCLC patients, for instance, the primary tumor and matched brain metastases differed in MVD, vessel maturity and VEGF expression [91].

Finally, regarding vessel normalization there is a need for a biomarker to evaluate this process in different tumor types and stages. Tumor perfusion is a potential biomarker to select patients who may benefit from combinations of antiangiogenic and other drugs. However, assessing the role of tumor perfusion requires innovative study designs and extensive clinical trials. A possible tool to eventually improve drug delivery to individual tumors, and thereby optimize outcomes of combination therapies, could be *in vivo* imaging of labeled drugs. This might clarify the interplay between vessel normalization and tumor drug delivery. Small clinical trials could be performed to visualize the effects of antiangiogenic drugs on the distribution of other labeled drugs, to provide serial information on whole body drug distribution, and to guide rational trial design for large combinatorial studies.
